# A novel canine histiocytic sarcoma cell line: initial characterization and utilization for drug screening studies

**DOI:** 10.1186/s12885-018-4132-0

**Published:** 2018-03-01

**Authors:** Marilia Takada, Maciej Parys, Emmalena Gregory-Bryson, Paulo Vilar Saavedra, Matti Kiupel, Vilma Yuzbasiyan-Gurkan

**Affiliations:** 1 Comparative Medicine and Integrative Biology, Michigan State University, East Lansing, MI 48824 USA; 2 Small Animal Clinical Science, Michigan State University, East Lansing, 48824 MI USA; 3 Pathobiology & Diagnostic Investigation, Michigan State University, East Lansing, MI 48824 USA; 4 Present address: Royal (Dick) School of Veterinary Studies and the Roslin Institute, Roslin, Midlothian, UK

**Keywords:** Histiocytic sarcoma, Dendritic cell, Cell line, Dog, Small molecule inhibitor

## Abstract

**Background:**

Histiocytic sarcoma is a rare disorder in humans, however it is seen with appreciable frequency in certain breeds of dogs, such as Bernese mountain dog. The purpose of this study was to fully characterize a novel canine histiocytic sarcoma cell line, and utilize it as a tool to screen for potential therapeutic drugs.

**Methods:**

The histiocytic sarcoma cell line was characterized by expression of cellular markers as determined by immunohistochemistry and flow cytometry techniques. The neoplastic cells were also evaluated for their capability of phagocytizing beads particles, and their potential to grow as xenograft in an immunodeficient mouse. We investigated the in vitro cytotoxic activity of a panel of thirteen compounds using the MTS proliferation assay. Inhibitory effects of different drugs were compared using one-way ANOVA, and multiple means were compared using Tukey’s test.

**Results:**

Neoplastic cells expressed CD11c, CD14, CD18, CD45, CD172a, CD204, MHC I, and vimentin. Expression of MHC II was upregulated after exposure to LPS. Furthermore, the established cell line clearly demonstrated phagocytic activity similar to positive controls of macrophage cell line. The xenograft mouse developed a palpable subcutaneous soft tissue mass after 29 days of inoculation, which histologically resembled the primary neoplasm. Dasatinib, a tyrosine kinase pan-inhibitor, significantly inhibited the growth of the cells in vitro within a clinically achievable and tolerable plasma concentration. The inhibitory response to dasatinib was augmented when combined with doxorubicin.

**Conclusions:**

In the present study we demonstrated that a novel canine histiocytic sarcoma cell line presents a valuable tool to evaluate novel treatment approaches. The neoplastic cell line favorably responded to dasatinib, which represents a promising anticancer strategy for the treatment of this malignancy in dogs and similar disorders in humans.

**Electronic supplementary material:**

The online version of this article (10.1186/s12885-018-4132-0) contains supplementary material, which is available to authorized users.

## Background

Histiocytic sarcoma (HS) is a highly aggressive hematopoietic neoplasm in humans and animals. In humans, HS accounts for less than 1% of all hematopoietic neoplasms [1, 2], affects all ages, but predominately adults, and involves lymph nodes and/or a variety of extranodal organs including skin, bone marrow, spleen, the gastrointestinal tract and the central nervous system [3, 4]. This malignancy is often approached with a combination of various modalities of treatment, including multi-drug chemotherapeutic protocols, radiation therapy, surgery, and bone marrow transplantation [5–7]. However, patients with HS in general respond poorly to any form of treatment, therefore this neoplasm carries a poor prognosis and has a high mortality rate. The low number of clinical cases is a limitation for an extended understanding of the pathogenesis of this disease in people, and restricts the investigation for novel and more efficacious forms of treatment.

In dogs, a similar disorder is commonly found in certain breeds, especially the Bernese mountain dog (BMD), in which the prevalence ranges from 15 to 25% of the population [8–12]. The genetic susceptibility to HS in dogs has been the focus of several investigations. Current published data of a population of BMD from US and Europe, suggests deregulation of the tumor suppressor genes *MTAP/CDKN2A*/*B* located within the region homologous to human chromosome 9p21 [13, 14]. Studying HS in dogs is of high importance as, similarly to people, it is a fatal disease characterized by rapid progression and high metastatic rate [15–18]. Thus dogs, with spontaneously occurring HS, are a crucial model for development of new approaches to treat this orphan disease in people. Affected canine patients also respond poorly to treatment. The currently most effective drug is *N*-(2-chloroethyl)-*N*′-cyclohexyl-N-nitrosourea (CCNU) that provides a reported response rate ranging from 29 to 46% for a median survival time of 85–96 days [16, 19]. A preliminary study using another cytotoxic drug, doxorubicin, showed a similar response rate of 46% for a median survival time of 93 days [20]. Additional alternatives of treatment have been investigated on small cohorts of dogs, including delivery of the human MHC non-restricted T-cell line TALL-104, frameless stereotactic radiosurgery, and chemotherapy using either paclitaxel or pegylated-liposomal doxorubicin; however, none has shown promising results [20–24].

Research groups have demonstrated the therapeutic potential of drugs as small molecule inhibitors against HS, based on in vitro studies [25–27]. Screening a large library of small molecules, Ito et al. successfully identified eight compounds of high potency (> 60% inhibition) at concentration of 100 nM [25]. Interestingly, only two drugs shared the same main target, showing the diversity of factors driving tumorigenesis in HS, and the potential benefit of drug combination hitting multiple targets. In humans, there is only a single report of four patients with HS treated with small molecule inhibitors, associated with the overexpression of targets for those drugs within the neoplasm, however efficacy could not be evaluated due to the small cohort of patients [28]. No published studies exist reporting the clinical use of small molecule inhibitors in dogs with HS.

We present here details of a novel HS cell line from a BMD, named the BD cell line, we have successfully established and demonstrated its utility in identification of potential novel therapeutic options [29]. Considering that to date, there is no human HS cell line available for research purposes, we believe that this cell line will provide an essential scientific tool for the study of this disease.

## Methods

### Origin of primary tumor

**Fig. 1:**
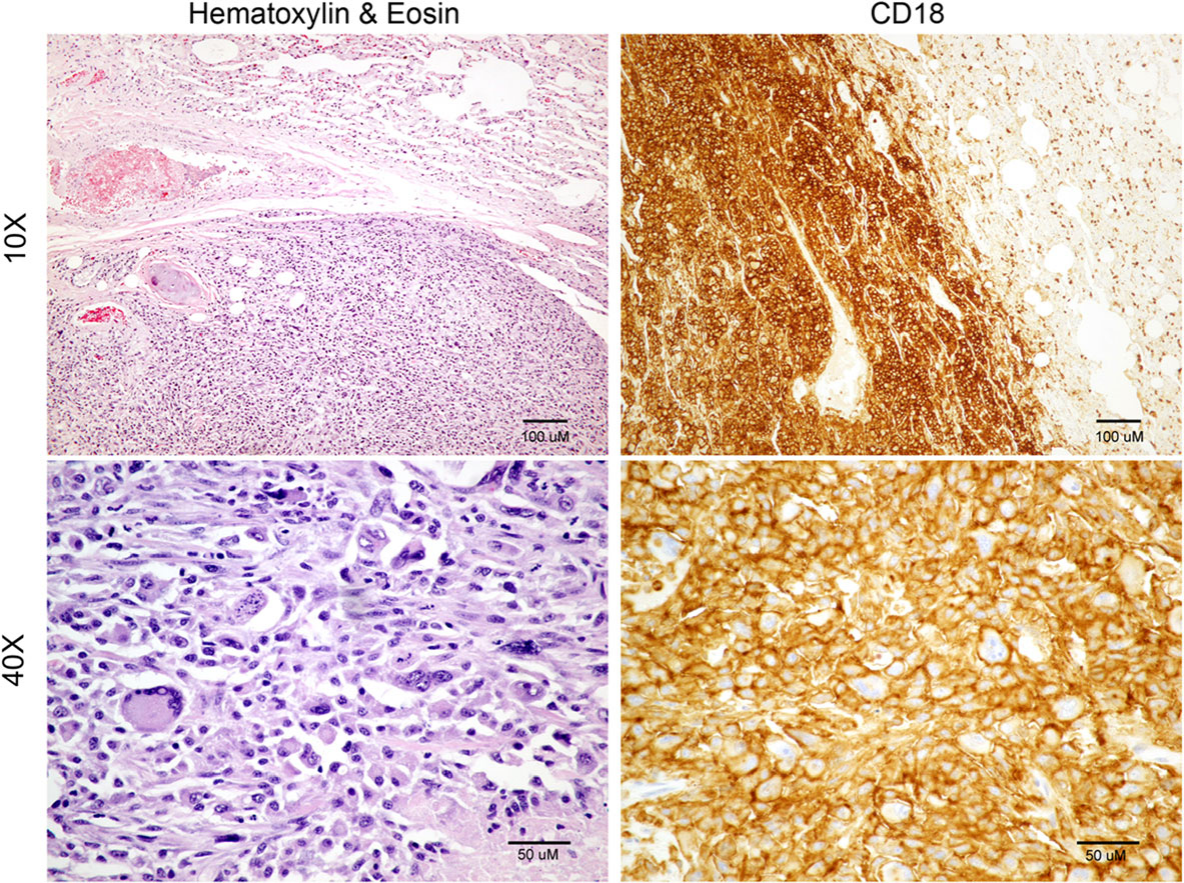
Histopathology and immunohistochemistry analysis of the primary neoplastic tissue. Sections of lung stained with hematoxylin and eosin (H&E) with a well-demarcated region of neoplastic cells replacing the normal pulmonary parenchyma. Neoplastic cells presented as a highly pleomorphic population of malignant histiocytic cells with marked anisocytosis and anisokaryosis. The cells contained large amounts of basophilic and typically mildly vacuolated cytoplasm. Multinucleation, megalocytosis and megalokaryosis were common features. One to several prominent nucleoli were present in most nuclei. Perimembranous expression of CD18 was strongly detected by immunohistochemistry

Fresh post-mortem tissue samples from both an abdominal and a pulmonary neoplasm were aseptically obtained from an 8-year-old spayed female BMD presented to the Michigan State University Veterinary Teaching Hospital. The diagnosis of HS was based on histopathology findings of the tumor and positive staining for CD18 by immunohistochemistry (Fig. 1) [8, 15, 30, 31].

Treatment with various chemotherapeutic agents was attempted over a year, including CCNU, doxorubicin and prednisone. The dog responded with only short periods of tumor remission until the owners opted for humane euthanasia due to the poor physical condition of the animal. Samples were collected immediately following euthanasia under informed owner consent.

### Preparation and maintenance of cell culture

The tissue samples were minced in small fragments with a surgical blade and placed in a solution of Hank’s Balanced Salt Solution with 1% collagenase (Sigma, St. Louis, MO) for cell disaggregation. After 40 min, the solution was transferred to a 70 μm cell strainer, and the filtered portion was plated in Roswell Park Memorial Institute 1640 medium (Life Technologies, Carlsbad, CA) supplemented with 10% heat-inactivated fetal bovine serum (FBS) (Life Technologies, Carlsbad, CA), 1% antibiotic-antimycotic 100X (Life Technologies, Carlsbad, CA), and 0.1% gentamycin (Life Technologies, Carlsbad, CA). The cell culture was then incubated at 37 °C in a humidified atmosphere of 5% CO_2_. For culture maintenance, the medium was thoroughly changed every 2–3 days. The cells were confirmed to be of canine origin and no mammalian interspecies contamination was detected based on results from CellCheck Plus test (IDEXX BioResearch, Columbia, MO). A genetic profile using a panel of microsatellite markers for genotyping is available and can be used in future monitoring, the detailed genotype is presented in Additional file 1.

### Cytology and immunohistochemistry

Concentrated cytospin slide of cell suspension was prepared, and stained with Diff-Quik for morphologic characterization by a board certified veterinary clinical pathologist. For immunohistochemistry analysis of the cell line, the cell pellet was fixed in 10% formalin for up to 24 h and transferred to 70% ethanol until embedding in paraffin. Sections from the paraffin block containing the cell pellet were deparaffinized in xylene and rehydrated in ethanol at different concentrations. Hydrogen peroxide 3% was used to neutralize endogenous peroxidases.

Antigen retrieval of formalin-fixed, paraffin embedded tissues was performed on the PT Link (Dako North America), using the EnVision FLEX Target Retrieval Solution, Low pH (Dako North America) for 20 min. For immunolabeling with the primary antibodies (CD3, CD18, CD79a and CD204), sections were processed with the Autostainer Link 48 (Dako North America, Carpinteria, CA) using the EnVision Flex+ detection system (Dako North America), and the immunoreaction was visualized with 3,3′-diaminobenzidine substrate (DAB) (Dako North America) and sections were counterstained with haematoxylin. For immunolabeling with antibody vimentin, the Discovery Ultra Automated Staining system (Ventana Medical Systems, Inc., Tucson, Arizona) was used with UltraMap alkaline phosphatase red detection system (Ventana Medical Systems, Inc) with a red chromogen. Antigen retrieval was achieved using the Ventana medical System antigen retrieval solution CC1 (Ventana Medical Systems, Inc., Tucson, Arizona) for 60 mins. Details of the antibodies are available in Additional file 2.

### Flow cytometry

BD cells harvested from cell culture were labeled with the following monoclonal antibodies: anti-canine CD3 (CA17.2A12, Serotec), anti-canine CD11c (CA11.6A1, UC Davis/NIH NeuroMab Facility), anti-bovine CD14 (MM61A, WSU MAC), anti-canine CD21 (CA2.1D6, Serotec), anti-canine CD45 (YKIX716.13, Serotec), anti-canine CD79a (HM57, LS Bio), anti-bovine CD172a (DH59B, WSU MAC), anti-canine MHC II (YKIX334.2, eBioscience), and anti-bovine MHC I (MHC CL I, WSU MAC). Isotype matched control antibodies were used to exclude non specific binding. The flow cytometers BD LSR II and BD Accuri C6 (BD Bioscience, Bedford, MA) were used for analysis. For the stimulation studies, BD cells were treated with either LPS 50 ng/ml (Sigma Aldrich, St. Louis, MO) or IFNγ 50 ng/ml (Peprotech, Rocky Hill, NJ), for 24 and 48 h. Details of antibodies are listed in Additional file 2.

### Phagocytosis assay

The phagocytic and endocytic properties of the established cell line were evaluated using 2% pHrodo™ *E. coli* Bioparticles® (Life Technologies, Carlsbad, CA). Using a 24-well plate, 100,000 cells were plated per well and left overnight. Culture medium was removed and replaced by 2% pHrodo™ *E. coli* Bioparticles® diluted in Live Cell Imaging Solution (Life Technologies, Carlsbad, CA) for 1.5–2 h before imaging. Confocal images were obtained using Leica TCS SPE confocal system (Leica Microsystems, Buffalo Grove, IL) on excitation wavelength of 460 nm. Commercially available murine macrophage cell line J774.A (ATCC® TIB-67™), a canine HS cell line DH82, derived from a macrophage derived sarcoma, hemophagocytic HS (ATCC® CRL-10389™), and canine fibroblasts isolated from the tunica albuginea were used for functional comparison purposes.

### Neoplastic cell growth and characterization in a xenograft mouse

In order to evaluate the ability of the cells to form tumor in vivo, 1 × 10^6^ neoplastic cells were injected into one ten-week old female mouse of NOD scid gamma strain (NOD.Cg-Prkdc^scid^ Il2rg^tm1Wjl^/SzJ, The Jackson Laboratory, Bar Harbor, ME). One million cells were suspended in 100 μl of Dulbecco’s Modified Eagle Medium (Life Technologies, Carlsbad, CA) with 10% FBS, and mixed with BD Matrigel™ Matrix HC in 1:1 ratio (BD Biosciences, San Jose, CA). The cell suspension was then inoculated subcutaneously into the left flank of the mouse under anesthesia.

The tumor growth in the inoculated mouse was monitored daily using calipers, until the tumor measured close to 10 mm in diameter as this was one of our humane endpoints. The mouse was sacrificed using carbon dioxide gas, and a full necropsy evaluated the presence of metastases into other organs. Tissues that had macroscopic changes were fixed in 10% formalin, routinely processed, and embedded in paraffin wax. Paraffin sections were stained with H&E for microscopic examination. For further characterization of the neoplasm, immunohistochemistry for CD18 was performed on paraffin sections.

### Drug-screening assays

For the drug-screening assays, we used both the BD cell line, and the DH82 (CRL-10389™ - ATCC®) cell line, established from a golden retriever with hemophagocytic HS. In total, 13 drugs (Table 1) were tested from stock solutions prepared with the appropriate solvent as indicated, stored at − 20 °C, and protected from light. Serial dilutions of each drug were made from the stock solutions in culture medium immediately before adding to the cells in such a way that the solvent concentration was always < 1%. Each compound was tested at different concentrations in order to bracket the corresponding IC_50_ (concentration of drug necessary to inhibit the cell growth by 50%).

**Table 1:** List of drugs from drug-screening assay with their respective solvent, and main known targets

Name	Solvent	Main Targets [65–71]
Dasatinib	DMSO	ABL, PDGFR, KIT, SRC
Erlotinib	DMSO	EGFR
Gefitinib	DMSO	EGFR
Imatinib	DMSO	ABL, PDGFR, KIT
Masitinib	DMSO	c-KIT, PDGFRα, −β, Lyn, FGFR3, FAK pathway
Nilotinib	DMSO	ABL, PDGFR, KIT
Toceranib	DMSO	VEGFR2, PDGFRβ, c-KIT
Sorafenib	DMSO	VEGFR2, PDGFR, KIT, FLT3, BRAF
Sunitinib	DMSO	VEGFR, KIT, PDGFR, RET, CSF1R, FLT3
Tozasertib	DMSO	SRC, GSK3, FLT3, JAK2, BCR-ABL
CCNU	Ethanol	Alkylation and cross-linking of DNA
Cladribine	DMSO	Purine analogue
Doxorubicin	Saline	Inhibits DNA topoisomerase II, induces DNA damage and apoptosis

### Viability assay and data analysis

For viability assay, DH82 and BD cell lines were seeded on 96-well plates with 7500 cells/well and 12,000 cells/well, respectively. After a 24-h incubation time, the cell culture medium was replaced with 100 μl of complete medium with drug, and cells were then incubated for 72 h. Subsequently, the viability of the cells was analyzed using a CellTiter 96® AQueous Non-Radioactive Cell Proliferation Assay (MTS) (Promega, Madison, WI). The formazan product was measured using EnVision® Multimode Plate Reader (PerkinElmer, Waltham, MA) at a wavelength of 490 nm. Each experiment was run in triplicate.

The background absorbance was subtracted from the absorbance values generated by the cells exposed to drugs. The effect on cell viability caused by each drug was calculated as follows: viability (%) = [1-(A-B)/(C-B)] × 100, where A is the response with drug, B is the background response with no drug, and C is the response with vehicle. The absorbance generated by the “cells alone” control was denoted as AbsIC_100_ (100%) and the absorbance generated by water control was denoted as AbsIC_0_ (0%). The calculated percentage at each (log10) drug concentration was then plotted using GraphPad Prism 5 software nonlinear regression curve fitting (PRISM 5, GraphPad Software, La Jolla, CA). The IC_50_ was determined as the drug concentration corresponding to the value of the mean between 0 and 100% viability. “Cells alone” control was treated with the vehicle DMSO 1%, the same concentration used to the cells exposed to the drugs.

### Drug combination assays

Both HS cell lines were counted and seeded in 96-well plates as described in *Viability assay and data analysis* section. Each cell line was incubated with 5 different concentrations of dasatinib with or without doxorubicin. The concentrations of dasatinib were defined to bracket the IC_50_ for each cell line, and the concentration of doxorucibin was fixed with the concentration necessary to inhibit cell viability by 70%. Cell viability was measured by MTS proliferation assay after 72 h of incubation. Each experiment was run in triplicate.

### Western blotting

BD cells were treated with either vehicle (0.1%) or dasatinib for 4 h. Cells were lysed using CellLytic M (Sigma-Aldrich) supplemented with 1% protease inhibitor and phosphatase cocktail inhibitor (P8340/P5726 – Sigma-Aldrich). A total of 40 μg of protein per lane were separated by Novex NuPAGE SDS-PAGE (4–12%), and transferred to polyvinylidene difluoride membrane. Membranes were blocked with 5% BSA in TBS buffer, and probed overnight with monoclonal rabbit anti-human antibodies against phospho-SRC (44-660G, Thermo Fisher) or SRC (2109, Cell Signaling), and monoclonal mouse anti-human β-actin (8H10D10, Cell Signaling). Secondary antibodies include IRDye 800CW goat anti-mouse and 680RD goat anti-rabbit (LI-COR). Infrared fluorescence was detected using Odyssey Imaging System (LI-COR), and analyzed using Image Studio™ Lite software (LI-COR).

### Statistical analysis

Comparison of means of the cell viability rate among groups of different drug concentrations was done by one-way ANOVA, followed by Tukey’s multiple means comparison test. In order to determine whether the addition of dasatinib to doxorubicin increased the anti-proliferative effect, two-way ANOVA was utilized. Statistical analyses were performed using GraphPad Prism 5 software (PRISM 5, GraphPad Software, La Jolla, CA). Differences were considered statistically significant when *p* < 0.05.

## Results

### Characteristics of BD cell line

**Fig. 2:**
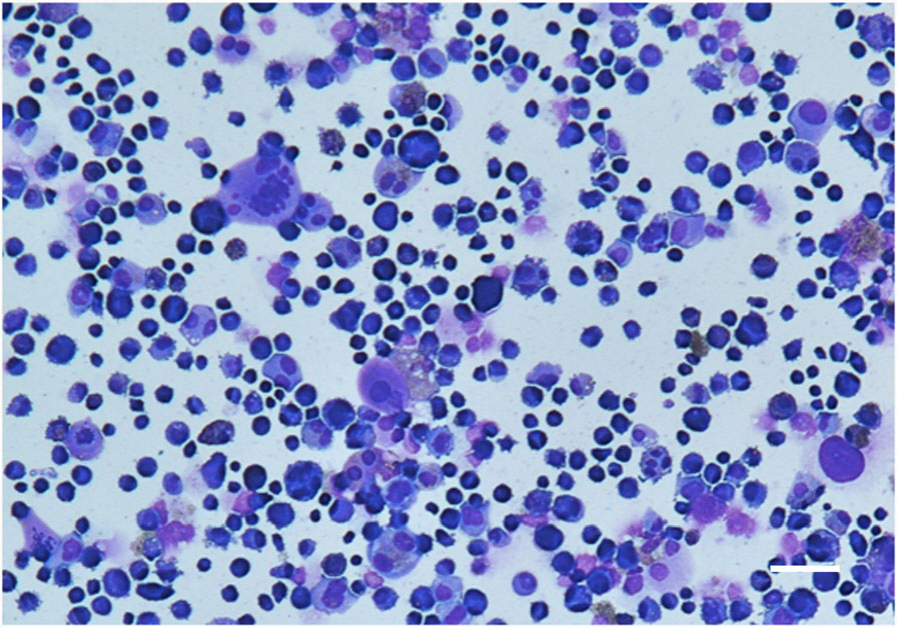
Image of BD cell cytospin stained with Diff-Quik showing a population of pleomorphic neoplastic round cells with marked anisocytosis and anisokaryosis with numerous binucleated and multinucleated cells. Calibration bar: 50 μm

Neoplastic cells in culture grew satisfactorily in 10% FBS without the addition of specific growth factors with the vast majority of the population growing as adherent and non-clustering cells. BD cell line was maintained in tissue culture for a minimum of 50 passages over 12 months, with a doubling time of approximately 36 h. Cytospins stained with Diff-Quik from cells harvested from cell culture showed the presence of significant cellular pleomorphism, marked anisocytosis and anisokaryosis. Giant cells and multinucleation were frequently observed (Fig. 2).

**Fig. 3:**
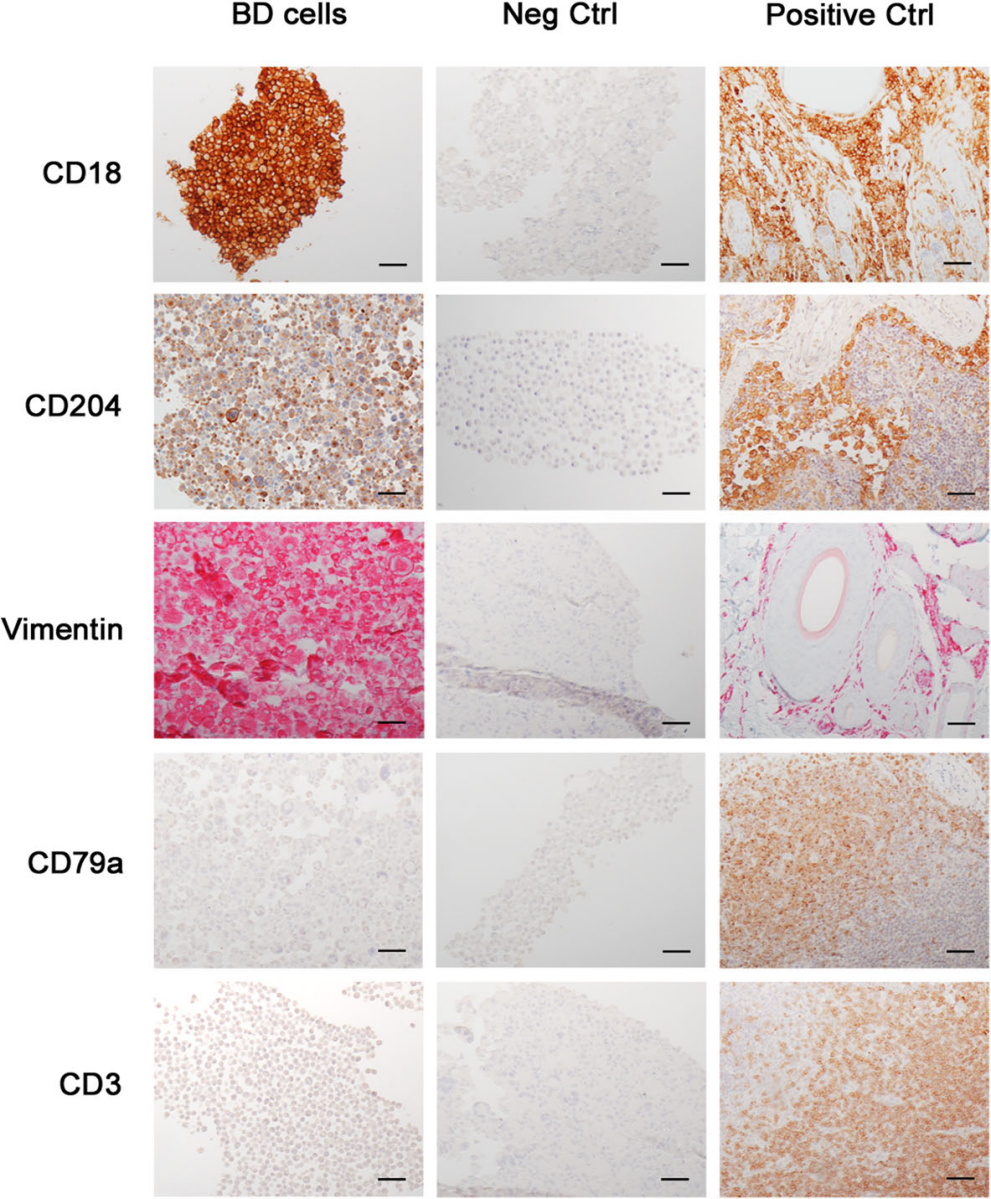
Immunohistochemistry of BD cell line showing strong expression of CD18, vimentin, and CD204, while being negative for CD3 and CD79a expression (left column). Middle and right columns are negatitve control and positive control sections, respectively. For CD18, CD204, CD3 and CD79a antibodies, DAB chromogen was used; and for vimentin antibody, alkaline phosphatase red chromogen. Calibration bar: 50 μm

**Fig. 4:**
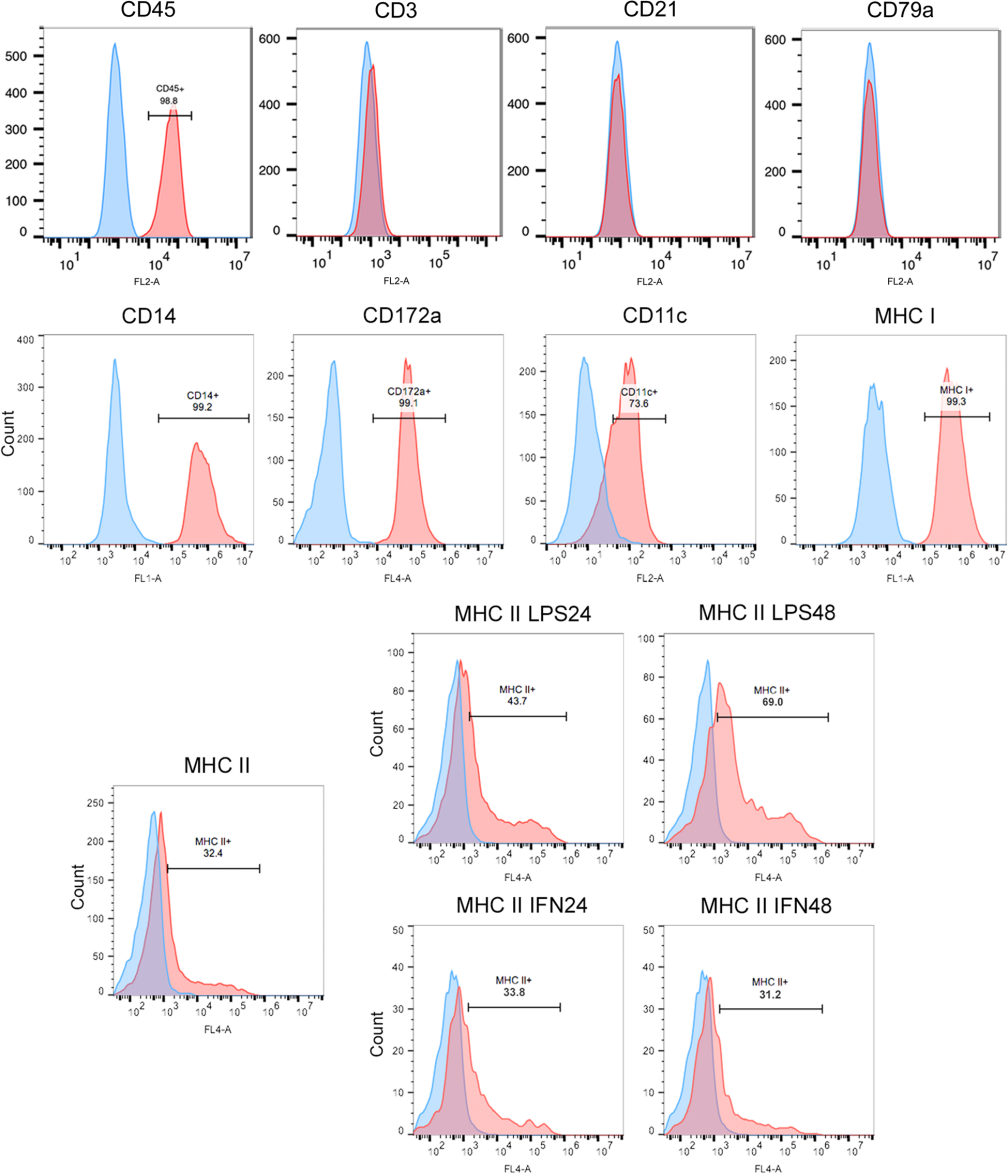
Flow cytometry results for expression of CD45, CD3, CD21, CD79a, CD14, CD172a, CD11c, MHC I, and MHC II. Expression of MHC II was also evaluated on cells treated with either LPS or IFNγ for 24 and 48 h. (BD cell line shown in red, isotype control in blue)

The established cell line showed lack of expression of both CD3 and CD79a, and positive expression of CD18, CD204 and vimentin on immunohistochemistry, consistent with features observed initially in the primary neoplasm (Fig. 3). The positive expression of myeloid marker CD45 and the lack of expression of lymphoid markers CD3, CD21 and CD79a were confirmed by flow cytometry. BD cell line expressed CD11c, CD14, CD172a and MHC I at a high level, and MHC II at a lower level (Fig. 4). MHC II expression was not affected by the stimulation with IFNγ, however it was significantly increased under the stimulation with LPS over time.

### Phagocytic properties of BD cell line

**Fig. 5:**
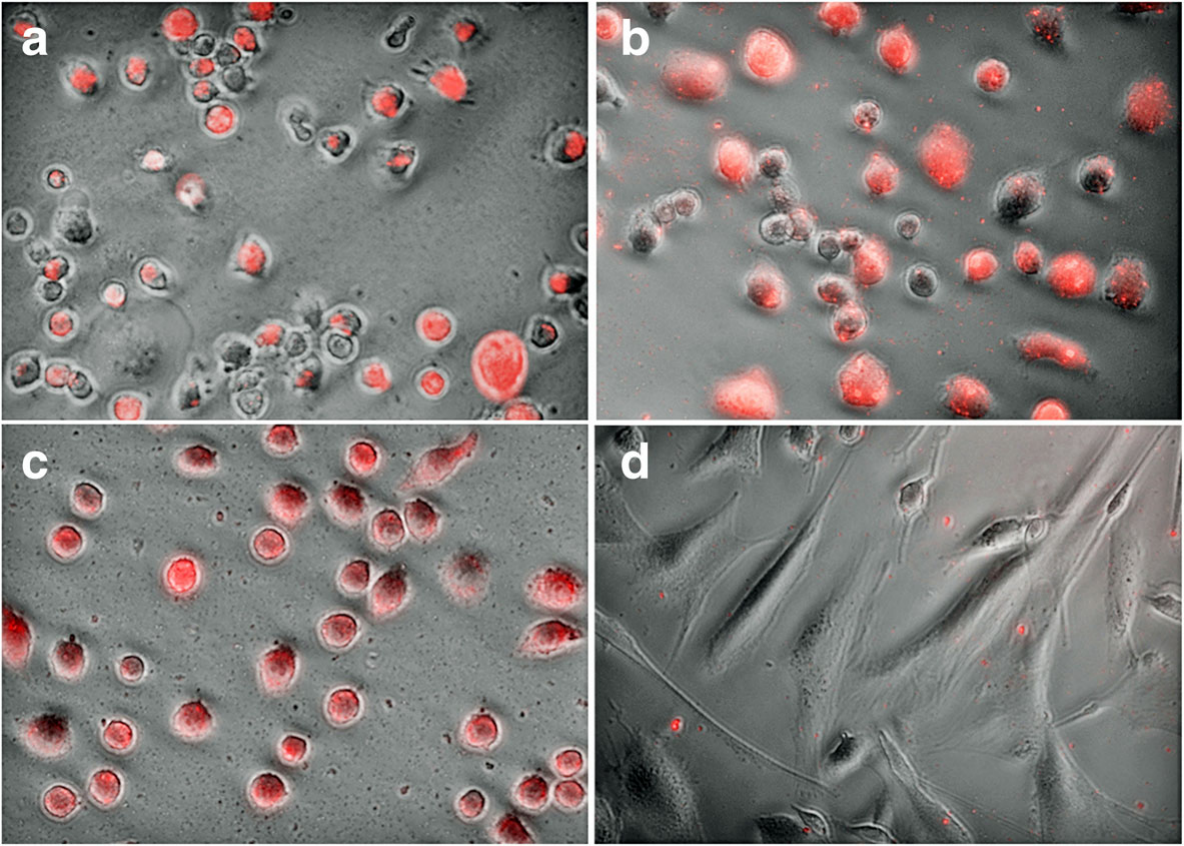
PHrodo Phagocytosis assay with strong uptake of red particles by the (**a**) BD cell line, the (**b**) canine HS cell line DH82, the (**c**) murine macrophage cell line J774A.1, and weak uptake by (**d**) canine fibroblasts

For the phagocytosis assays, the majority of cells from BD cell line were capable of phagocytizing the bioparticles, demonstrated by fluorescent intracellular signal on most of the cells (Fig. 5a). Both the murine macrophage cell line J774A.1 and the canine HS cell line DH82 demonstrated a high capability of phagocytosis, as expected (Fig. 5b and c). In contrast, we observed the presence of the bioparticles inside only a few canine fibroblasts (Fig. 5d).

### Experiment with xenograft mouse

The xenograft mouse developed a palpable soft tissue mass of 5 mm of diameter detected at the injection site 29 days after injection. The tumor increased rapidly in size over 6 days until it reached 10 mm of diameter, when the mouse was humanely euthanized. A full necropsy revealed a large lobulated subcutaneous mass at the injection site measuring 25 × 15 × 10 mm, showed in more detail in Additional file 3. The neoplasm had locally invaded the musculature of the abdominal wall dorsally to the tumor. No other significant macroscopic changes were observed in other organs.

### Evaluation of inhibitory effects of drugs on the growth of canine HS cells

**Fig. 6:**
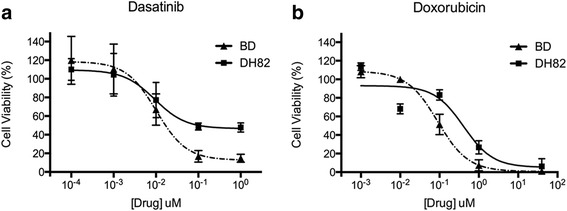
Dose-dependent antiproliferative activity of doxorubicin (**a**) and dasatinib (**b**) against BD and DH82 cells after 72 h of treatment. Cells treated with vehicle DMSO were used as control cells

The inhibition of cell growth by the drugs was variable between the two HS cell lines, BD and DH82. Dose-response curves were generated based on the cell viability at various concentrations of drug, from which IC_50_ values were determined. The results of IC_50_ (Table 2) values indicated that across 13 drugs tested, only dasatinib and doxorubicin were capable of inhibiting the growth of HS cells, within a pharmacologically relevant concentration. Both drugs exhibited statistically significant inhibitory effects on cell growth in a dose dependent manner using one-way ANOVA analysis (*p* < 0.0001). The IC_50_s of dasatinib were 10 and 9 nM, and of doxorubicin were 90 and 412 nM for BD and DH82 cell lines, respectively (Fig. 6). These concentrations were within the known tolerable plasma concentration values (Table 2). The maximum achievable plasma concentrations of the drugs were based on the plasma concentration values of the no-observed-adverse-effect level (NOAEL), or on the level of the therapeutic dosage described in the veterinary and human medicine literature. When available, the plasma concentration values encountered for dogs were used over those for humans.

**Table 2:** Results from drug screening and referenced achievable plasma concentrations

Drug	IC_50_ (μM)		Achievable concentrations in plasma (based on the literature)				
	BD	DH82	[plasma] (μM)	Species	Dose	Effect	Ref.
Dasatinib	0.01	0.009	0.3	dog	3 mg/kg	NOAEL	[72]
Erlotinib	3.2	4.3	6.9	dog	150 mg	TD	[73]
Gefitinib	30.7	46.4	2.6	dog	100 mg/kg	NOAEL	[74]
Imatinib	34	39.2	0.66	dog	10 mg/kg	NOAEL	[75]
Masitinib	15.7	39.1	1.5	dog	10 mg/kg	TD	[76]
Nilotinib	29.9	26.2	1.4	dog	20 mg/kg	NOAEL	[77]
Toceranib	1.9	1.7	0.1	dog	3.25 mg/kg	TD	[78]
Sorafenib	36.3	13.6	13.3	human	400 mg	TD	[79]
Sunitinib	4.5	17.9	0.12	human	50 mg	TD	[80]
Tozasertib	7.7	1.3	0.27	human	8 mg/m^2^	TD	[81]
CCNU	105	139.5	4.2	human	130 mg/m^2^	TD	[69]
Cladribine ^a^	64	222	0.028	human	0.09 mg/kg	TD	[82]
Doxorubicin ^a^	0.09	0.41	1.13	dog	30 mg/m^2^	TD	[83]

^a^ Compounds administered intravenously

NOAEL: no-observed-adverse-effect level

TD: therapeutic dosage

Results of IC_50_ (nM) values of drugs for each HS cell line, and associated values of achievable plasma concentration described in the veterinary and human medicine literature.

### Exploring synergistic combinations of drugs

**Fig. 7:**
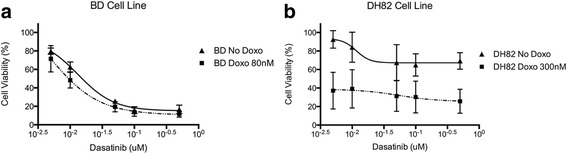
The effect of dasatinib on cell viability administered alone and in combination with doxorubicin on BD (**a**) and DH82 (**b**) cell lines

We next assessed the resulting cytostatic effect caused by increasing concentrations of dasatinib combined to doxorubicin at a fixed concentration close to the correspondent cell line’s IC_50_. When BD cell line was treated with the drug combination for 72 h, there was no significant increase in the antiproliferative activity in comparison to the cells treated with dasatinib alone. On the other hand, dasatinib and doxorubicin combination significantly increased the inhibitory effect on the growth of DH82 cells (*p* = 0.0003) (Fig. 7).

### Effect of dasatinib on SRC activity

**Fig. 8:**
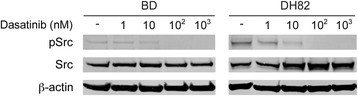
The effect of dasatinib on expression of phospho-SRC on BD and DH82 cell lines

The expression of total SRC and phopho-SRC was evaluated on cells treated with escalating concentrations of dasatinib. We observed a decrease in phospho-SRC but not in total SRC in both cell lines, BD and DH82, after 4 h of treatment. Expression of phosphor-SRC was minimal at the concentration of 100 nM of dasatinib (Fig. 8).

## Discussion

The use of the naturally occurring histiocytic sarcoma in the dog as a translational model for human HS represents a valuable opportunity to further understand this malignancy, and to find better tools for treatment. In this article we have presented the initial characterization of a novel HS cell line - BD cell line - and investigated potential novel treatment approaches.

Histiocytic sarcomas are frequently associated with a high level of cellular pleomorphism, therefore, the definitive diagnosis is determined by a pattern of expression of markers specific for histiocytes. Although, several markers have been used to characterize human HS including CD163, CD68, CD11c, lysozyme, and CD14 [4, 32, 33], two different markers were validated for HS tumors in dogs: CD18 (integrin beta chain beta 2) and CD204 (class A macrophage scavenger receptor) [8, 15, 30, 31, 34]. The novel BD cell line showed positive expression of CD18 and CD204, consistent with the diagnosis of HS. The negative expression of CD3, CD21 and CD79a excluded lymphoid malignancies, while positive vimentin, a type III intermediate filament protein, confirmed the mesenchymal origin, and positive CD45 confirmed the myeloid origin. Interestingly, the stimulation of BD cells with LPS, a lipopolysaccharide component of Gram-negative bacteria walls, resulted in increased expression of MHC class II. A similar increase in MHC Class II expression was reported when murine dendritic cells were exposed to LPS, including CD14 positive dendritic cells [35, 36]. CD14 expression is mainly present in macrophages and monocytes, however it can also be found in DCs, where it has been proposed to play a role on the immunologic responses in DCs [37]. The same effect was not observed when the cells were stimulated with interferon-γ (IFNγ). Due to the fact that the majority of BD cells expressed MHC I and MHC II, we could confirm an antigen presenting cell profile. Both macrophages and DCs are antigen presenting cells that, despite their differences in biological functions, are only distinguished by a few surface markers. Candidate markers that have an increased specificity for DC and DC subsets have been identified in several studies [38–41]. Two DC-specific markers were tested in the present study, CD11c and CD172a, for which the vast majority of BD cells were positive. Together, these findings indicated a pattern of expression associated with a dendritic cell origin.

Dendritic cells are professional phagocytes that have an important role in processing antigens for adaptive immune recognition [42]. The majority of cells from the newly established cell line were able to phagocytize pHrodo E. coli bioparticles when coincubated for 2 h. In this assay, the bioparticles would only emit fluorescence once they are inside the phagosomes of the cells, where the pH is low. Therefore, this pH-sensitive fluorescent method permits a clear discrimination of where the particles are located in respect to the cells [43]. As a representation of positive controls for this assay, the two macrophage cell lines, J774.A and DH82, showed a high level of phagocytosis. In contrast, we could appreciate phagocytic activity in only a very small number of canine fibroblasts, which were used as negative control.

When transplanted to an immunodeficient xenograft mouse, BD cell line successfully grew as a palpable soft tissue mass after 29 days, a time-frame comparable to other tumor xenograft studies [44]. We were able to confirm that BD cell line is a tumorigenic cell line, and could potentially be useful for in vivo studies as an experimental tool to study histiocytic sarcoma. Further studies with a larger number of animals are warranted to truly establish the transplantability and metastatic potential. A few xenograft mouse models of canine HS have been successfully established and were used for the evaluation of their metastatic potential, and to study the phenomenon of resistance to CCNU-based treatment [45, 46]; however, none of these cell lines had originated from BMDs, the most frequently affected breed. To the best of our knowledge, across the many existent canine HS cell lines, only two others were reported to be originated from tumors of BMDs [47]; and these two were not part of their drug response report.

Results from the in vitro drug screening experiment showed a variable response between the different drugs, and across the cell lines. Across a total of 13 compounds, dasatinib and doxorubicin effectively inhibited the cell growth of both HS cell lines within a clinically tolerable and achievable plasma concentration according to the veterinary and human literature.

Doxorubicin was the only conventional chemotherapeutic drug that elicited a favorable response against the HS cell lines. Several studies have reported the use of doxorubicin for the treatment of histiocytic disorders, including HS [5, 6, 33, 48–51]. Due to the low number of cases, the effectiveness of doxorubicin could not be determined in those studies, especially as single agent, as the drug was invariably used in a drug combination protocol. Our results agree with previous in vitro studies that considered doxorubicin an effective drug against canine HS [20, 52]. The potential of doxorubicin for the treatment of dogs with HS was suggested by a preliminary clinical study, where the response to doxorubicin was comparable to the response to the drug with the best response reported to date, CCNU [20].

Our positive results with dasatinib are in accordance with results from a study by Ito et al., where dasatinib was effective against 4 out of 7 canine HS cell lines derived from various breeds. The calculated IC_50_ values from that study varied from 5.4 to 54.5 nM, while the average IC_50_ value in our study was 9.5 nM, clearly within their range [25]. Dasatinib is an multi tyrosine kinase inhibitor with multiple main targets, including the SRC family kinases (SRC, LCK, YES, and FYN), the BCR-ABL, and to a lesser extent, c-KIT, PDGFRβ and EPHA2 [53]. We demonstrated that dasatinib inhibited the activation of SRC, as revealed by decrease of p-SRC (phospho-SRC) in both cell lines, BD and DH82. We hypothesize that the downregulation of p-SRC could be associated with the inhibitory effect of dasatinib on the HS cell lines, however, as a multi-kinase inhibitor, many other molecular targets might also be affected. However, the SRC pathway is a major oncogenic driver involved in HS against which dasatinib and other novel compounds may be used. Although dasatinib has been investigated in many human cancers, its clinical therapeutic value for HS has never been documented. [54–58]. However, the use of other small inhibitors for the treatment of HS has been reported in one study where human patients were treated with imatinib, sorafenib and bevacizumab, based on the pattern of expression of key molecular targets [28]. Another set of studies reported two patients carrying a HS associated with a mutation in BRAF^V600E^ gene, most commonly seen in melanomas in humans [50, 59]. Neoplasms that are driven by this mutation are suitable for vemurafenib-based treatment, a B-Raf small molecule inhibitor. In fact, a human patient from one of the studies was treated with vemurafenib and experienced therapeutic response for a couple of months [59]. Although the efficacy of these drugs could not be evaluated due to the small cohort of patients, the identification of druggable targets in HS shows the value of targeted therapy for this disease.

We demonstrated that the combination of dasatinib and doxorubicin resulted in a favorable additive effect against one of the two HS cell lines, DH82 cell line. The association of small inhibitors with chemotherapeutic drugs has been strategized as a treatment with broader spectrum, which resulted in synergistic anticancer activity for many tumors [26, 60, 61]. Often the increased efficiency is accompanied with a dose reduction of the drugs, and consequently decreased treatment related side effects. The combination of drugs with different mechanisms of action should be considered as a relevant strategy to optimize the therapeutic effect of each inhibitor against HS.

## Conclusions

The present study was able to establish and fully characterize a DC-subtype HS cell line from a tumor in a BMD. This novel HS cell line represents a model available not only for the investigation of potential therapeutic drugs, but also for the studies of gene expression and genetic variability associated with HS. The existence of cancer cell lines has been a critical tool for the understanding of cancer biology and response to therapy. For that matter, panels of human cancer cell lines such as NCI60 and GDSC have become available for researches, so that the collective use of these resources would generate more efficiently information including genomics and drug sensitivity of these cell lines [62, 63]. Nevertheless, it is valuable to mention that cell lines are prone to genetic changes over time, and early passages should be a better model of the tumor of origin. More recently, a panel of canine cancer cell lines has been created covering the most important cancers seen in canine patients [64]. Due to the rarity of HS in humans, studies based on spontaneous HS in dogs can provide important and relevant translational understanding of this malignancy. Moreover, we also propose BD cell line as a useful system for studies where a cell line of dendritic cell origin can be of value.

## Additional files

Additional file 1: Genetic profile of BD cell line genotyped using of a panel of microsatellite markers. (XLSX 31 kb)

Additional file 2: Description of primary and secondary antibodies. (XLSX 27 kb)

Additional file 3: Xenograft tumor in a mouse. On necropsy, a large lobulated subcutaneous mass was present at the site of injection of tumor cells 35 days after transplantation (black arrow). (TIFF 7833 kb)

## Abbreviations

BMD: Bernese mountain dog
CCNU: *N*-(2-chloroethyl)-*N*′-cyclohexyl-N-nitrosourea
CD: cluster of differentiation
DAB: 3,3′-diaminobenzidine
DC: Dendritic cell
H&E: Hematoxylin and eosin
HS: Histiocytic sarcoma
IFNγ: Interferon-γ
MHC: Major histocompatibility complex
