# Supplementary figures and images for: A novel canine histiocytic sarcoma cell line: initial characterization and utilization for drug screening studies
Source: BMC Cancer. 2018 Mar 1;18:237. doi: 10.1186/s12885-018-4132-0 (PMC5831740; doi:10.1186/s12885-018-4132-0)

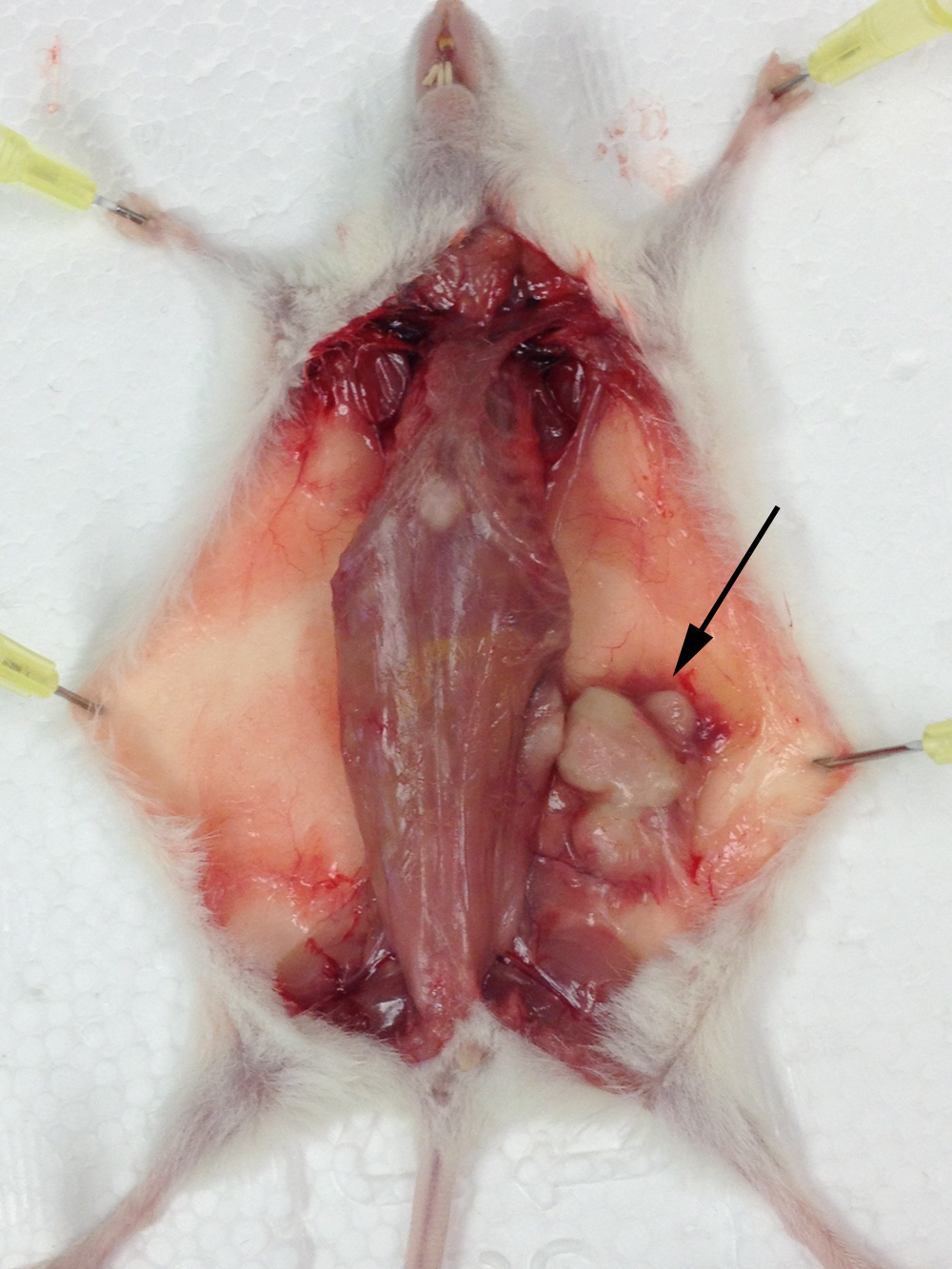

Supplement: Supplementary file 3 — Xenograft tumor in a mouse. On necropsy, a large lobulated subcutaneous mass was present at the site of injection of tumor cells 35 days after transplantation (black arrow). (TIFF 7833 kb) [file 12885_2018_4132_MOESM3_ESM.tif]
